# Mechanical behavior and constitutive modeling of CO_2_-foamed carbonated MgO-based solidified slurry under Unconsolidated Undrained (UU) triaxial conditions

**DOI:** 10.1371/journal.pone.0351078

**Published:** 2026-06-17

**Authors:** Ping Gao, Jinbo Xie, Qinglong You, Xi Du, Li Shao, Weihong Xie

**Affiliations:** 1 College of Building Engineering, Shanghai Zhongqiao Vocational and Technical University, Shanghai, China; 2 Department of Civil Engineering, School of Environment and Architecture, University of Shanghai for Science and Technology, Shanghai, China; Guizhou University of Engineering Science, CHINA

## Abstract

Geotechnical construction activities, such as shield tunneling and pile foundation construction, generate large amounts of high-water-content waste slurry, making its disposal and resource utilization challenging. This paper introduces a CO_2_-foamed carbonation solidification technique using a composite binder system made of reactive magnesium oxide (MgO), slag, and calcium carbide slag. The mechanical behavior of the lightweight carbonated slurry was investigated through Unconsolidated Undrained (UU) triaxial compression tests under various confining pressures. Quantitative results indicate that the reactive MgO dosage significantly governs strength evolution; an optimum MgO dosage of 10% yielded the highest peak stress of 190.8 kPa (at 50 kPa confining pressure), while the softening coefficient peaked at 0.46 with a 13% dosage. Regarding the CO_2_ foam impact, varying the foam content from 36% to 52% resulted in softening coefficients ranging between 0.20 and 0.50. The 36% foam dosage provided the optimal structural stability with a peak stress of 179 kPa, whereas excessive foam leads to greater pore connectivity, worsening strain-softening. Confining pressure significantly affects the material’s hardening and residual strength. Based on these findings, an improved constitutive model was developed using damage evolution and a modified Duncan–Chang framework, providing accurate descriptions of the material’s elastic, hardening, post-peak softening, and residual characteristics. This study advances our understanding of CO_2_-foamed carbonated MgO-based slurry, offering new insights for both solidification/stabilization and carbon sequestration of high-water-content waste slurry.

## 1. Introduction

As global urbanization accelerates, large-scale underground works such as shield tunneling and deep excavations have become routine. A severe associated challenge is the on-site generation of vast quantities of waste slurry. Characterized by extremely high water content, very low shear strength, and poor engineering stability, this slurry is difficult to handle, creating problems for both project management and environmental protection. Recent studies have emphasized the importance of characterizing physical properties, controlling swelling behavior, and employing advanced prediction methods to address the stability challenges of such problematic fine-grained soils [1–4]. Conventional approaches like haulage and landfilling incur high logistics costs due to the slurry’s moisture and bulk density, consume scarce land resources, and introduce long-term environmental and safety risks. There is a pressing need to shift from “passive disposal” to “active resource utilization” by pursuing sustainable routes that simultaneously lower bulk density (lightweighting) and deliver solidified products with excellent engineering performance [5–7].

In this context, reactive magnesia (MgO)-based cementitious materials have gained significant attention due to their environmental benefits. Unlike Portland cement (PC), which requires energy-intensive clinker production and releases large amounts of CO_2_, reactive magnesia cement (RMC) hardens through carbonation in CO_2_-rich environments [8–11]. Carbonation not only consumes but also permanently sequesters CO_2_, while producing hydrated magnesium carbonate (HMC) phases, such as nesquehonite and magnesite, which serve as high-strength cementitious materials. These dense carbonates fill pores and enhance the microstructure, leading to significant improvements in both early-age and long-term mechanical performance. This combination of “carbon sequestration” and “solidification” places RMC at the forefront of research on low-carbon geotechnical materials.

To address the high bulk density of waste slurry, foamed lightweight materials, such as lightweight foamed soil or foam concrete, offer a well-established solution [12–14]. These materials process adjustable density, high fluidity, and excellent workability, making them suitable for backfill, subgrade replacement, and load reduction. A more innovative approach combines lightweighting with carbonation: by using CO_2_ directly as the foaming agent or adopting CO_2_-stabilized foams, the “foaming–carbonation” processes are synchronized [15–17]. This “process-as-reactant” concept not only reduces density but also creates a vast gas–solid interface within the slurry. CO_2_ can permeate foam films and interconnected pores, enabling uniform and efficient in situ carbonation and sequestration throughout the material, thus overcoming the surface-limited challenges of conventional external-diffusion carbonation. Further studies demonstrate that CO_2_ foams can be effectively stabilized through surfactant modification or sol–gel techniques, ensuring stability in alkaline slurries and promoting uniform carbonate precipitation.

To further reduce costs and embodied carbon, a “waste-treats-waste” approach can be incorporated into the composite binder. Industrial solid wastes, such as slag, steel slag, and calcium carbide slag (CCS), offer promising components. In particular, CCS acts as a low-cost alkali activator due to its calcium-rich, highly alkaline nature [18–20]. When properly activated, CCS participates in a synergistic hydration and carbonation process with MgO and aluminosilicate materials (e.g., slag), forming multi-component cementitious phases, including C–S–H, M–S–H, and hydrated magnesium carbonates (HMCs). These reactions refine the pore structure and improve strength. The integration of alkali activation of industrial wastes, MgO carbonation-hardening, and CO_2_ foaming offers the potential for simultaneous improvements in mechanical performance, sequestration efficiency, and lightweighting. However, the underlying mechanisms within this coupled “MgO–solid waste–CO_2_ foam” system are complex, and the interactions between reaction pathways, competition and synergy among product phases, and the evolution of pore structure at different scales are still not fully understood or quantitatively explored [18–21].

From an engineering mechanics perspective, this carbonated, porous, cemented material demonstrates significant strain-softening behavior. Its complete stress–strain response typically includes distinct stages: elastic, non-linear hardening, peak, post-peak softening, and residual plateau stages. Factors such as confining pressure, foam content (a resulting material porosity), binder proportion, and carbonation degree play key roles in determining peak strength, softening rate, and residual strength. However, systematic triaxial data on the specific “CO_2_ foam–MgO–solid waste” system remain limited, particularly from UU tests. The evolution of the softening coefficient and the parametric characterization of residual strength under varying confining pressures remain poorly understood, which complicates the robust assessment of stability under complex stress paths. Accurate prediction is crucial for design, but conventional empirical or semi-empirical constitutive models, such as the Duncan–Chang model, struggle with materials that exhibit strong softening, damage accumulation, and a pronounced residual stage. These models often fail to capture post-peak degradation, which could lead to non-conservative designs. Therefore, a new constitutive relationship that incorporates damage variables, modulus degradation, and residual strength is urgently needed [22,23].

Accordingly, this study employs a composite binder comprising reactive MgO, slag, and calcium carbide slag, utilizing CO_2_ foam as the dual-function foaming and carbonation medium. Lightweight, carbonated, solidified-slurry specimens are prepared following the optimized mix proportions and fabrication procedures proposed by Shao et al. [10]. Through systematic UU triaxial shear tests, we investigate strength evolution, softening mechanisms, and residual behavior under multiple confining pressures, across varying MgO dosages and CO_2_ foam volume fractions (i.e., target densities). Building on these results, we develop an improved constitutive model that accurately represents the elastic–plastic stage, post-peak softening, and residual plateau. The work addresses critical gaps in understanding the mechanical response of CO_2_-foamed, MgO-solidified, industrial-waste–assisted carbonation systems under engineering loading, and offers theoretical and methodological guidance for rapid solidification, lightweighting, and resource utilization of high-water-content waste slurry.

## 2. Materials and experimental program

### 2.1 Experimental materials

**Fig 1:**
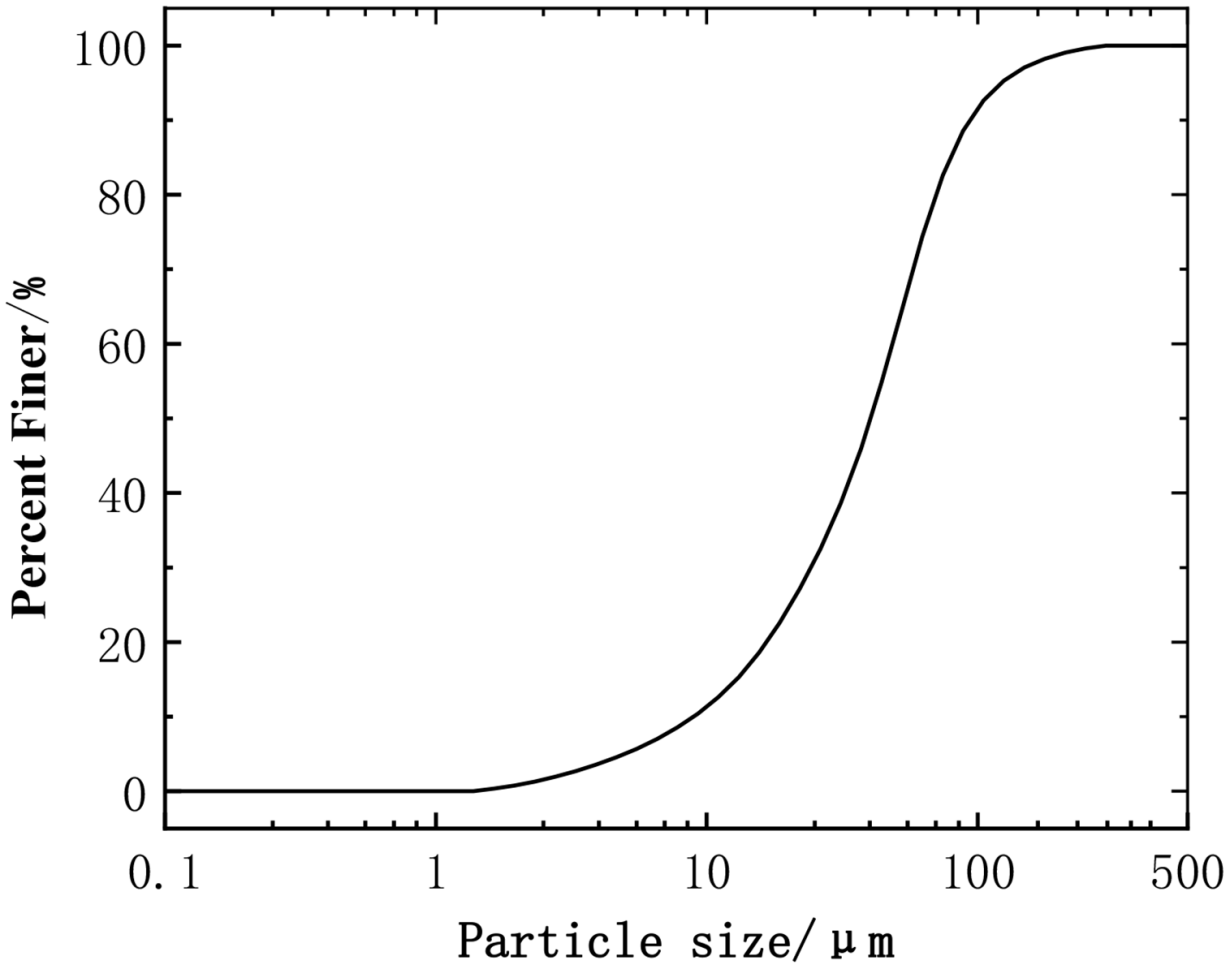
Particle analysis curve of waste slurry.

The waste slurry used in this study was obtained from bored pile construction slurry at a construction site in the West Bund Camp, Xuhui District, Shanghai. The slurry appeared grayish-black and was characterized by a high water content. It was sampled, transported, and stored in sealed barrels to prevent moisture evaporation. In accordance with GB/T50123-2019 [24] and (DL/T 5815−2020 [25], its water content, pH value, liquid and plastic limits, and specific gravity were determined. The specific physical properties are detailed in Table 1. The particle size distribution of the soil sample was measured using a laser particle size analyzer. The results of the particle analysis are presented in Fig 1.

**Table 1:** Physicochemical properties of waste slurry.

Water Content (%)	pH	Liquid Limit (%)	Plastic Limit (%)	Specific Gravity (g/cm^3^)
197.7	7.28	31	18.5	1.18

The composite binder system consisted of reactive magnesium oxide (MgO), slag powder, and calcium carbide slag. The chemical composition of the reactive MgO is provided in Table 2.

**Table 2:** Chemical composition of reactive magnesium oxide.

Component	MgO (%)	CaO (%)	Fe (%)	Al (%)	HCl Insoluble (%)
Content	98.00	0.60	0.02	0.01	0.10

The calcium carbide slag powder used in this study was a high-quality (Grade I) fine powder, gray in color, and exhibited high reactivity. It had a particle size distribution of approximately 150–400 mesh and a density of 1.5 g/cm^3^. The specific chemical composition is presented in Table 3.

**Table 3:** Chemical compositions of calcium carbide slag and slag powder (wt%).

Component	Calcium Carbide Slag	Slag Powder
CaO	6.56	34
SiO_2_	3.5	34.5
Al_2_O_3_	1.62	17.7
MgO	—	6.01
Fe_2_O_3_	—	1.03
SO_3_	—	1.64
Ca(OH)_2_	92.7	—
H_2_O	0.5	—

The S95 grade slag powder selected for this study had a density of 2.8 g/cm^3^, a flowability ratio of 98%, a loss on ignition (LOI) of 0.84%, and a water content of 0.45%. It appeared grayish-white. Its specific chemical composition is detailed in Table 3.

The slag activity index is a crucial criterion for assessing the quality of the slag powder. It is determined by preparing mortar specimens with a 1:1 mass ratio of cement to slag powder and testing their 7-day (7d) and 28-day (28d) compressive strengths, which are then calculated as a ratio relative to the compressive strengths of pure cement mortar specimens at the same ages. The slag powder used in this paper was Grade S95, with a 28d activity index of 98.5%. This 28d activity index (exceeding 95%) signifies that the compressive strength of the mortar containing 50% slag powder is not less than 95% of that of the pure cement mortar control specimens at the 28-day curing age.

The anhydrous calcium sulfate (CaSO_4_) used in this study was of analytical reagent (AR) grade. Its detailed technical specifications are provided in Table 4.

**Table 4:** Technical specifications of anhydrous calcium sulfate.

Anhydrous calcium sulfate content (%)	Ammonium content (%)	Drying vector (%)	HCl insoluble (%)	Alkali metals and magnesium (%)
97.00	0.03	1.00	0.05	0.50

Polyacrylamide (anionic, analytical reagent grade), hereafter referred to as PAM, was utilized. It possessed a PAM content of ≥ 90% and a degree of hydrolysis of 30%. The material, a white crystalline solid, had a pH range of 5.0–7.0 and an anionic value of 1.2–1.6. It is characterized by its good flocculation performance. The CO_2_ gas used was high-purity, food-grade CO_2_, with a purity of ≥99%.

**Fig 2:**
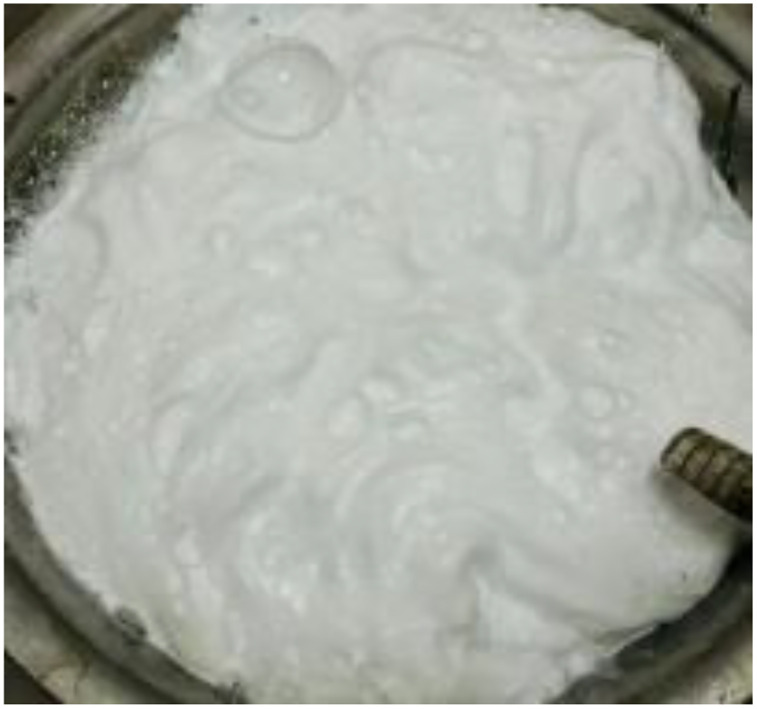
CO_2_ foam used in the experiment.

Foaming Agent Foaming agents are generally categorized into four types: rosin-based, animal/plant protein-based, surfactant-based, and composite-type. The agent selected for this study was a composite-type, exhibiting a high foaming ratio and good foam stability. The density of the foam generated was 40 kg/m^3^ when foamed with air, and 80 kg/m^3^ when foamed with CO_2_. The CO_2_ foam is shown in Fig 2.

### 2.2 Specimen preparation and carbonation

This study prepared specimens based on the optimal mix proportions derived from the preliminary experiments by Shao et al. [10]. The specific procedure was as follows:

The waste slurry retrieved from the construction site was pre-treated (e.g., stirred and oven-dried) and then stored in sealed containers. Prior to testing, a high-water-content slurry was prepared at a preset water content of 200%. Based on the target proportions, the solidifying agents were weighed and the powders were blended thoroughly; they were then added to the prepared high-water slurry in two slow batches, with the mixture stirred for 3 minutes after each addition to ensure even mixing. This ensured uniform dispersion throughout the slurry.

CO_2_ foaming began with pre-treating the alkaline foaming agent, because it reacts readily with CO_2_ and CO_2_ dissolves in the solution, both of which can make the foam unstable and deflate quickly, so pre-treatment was essential; the agent was diluted 1:40 and sparged with CO_2_ at 200 kPa until a stable foam formed; the gas inlet valve was then closed and the solution rested for 20 minutes to allow full reaction, while the pressure in the foam generator was kept steady—if any drop occurred, the inlet was reopened to replenish CO_2_ and hold the set value; once stability was achieved, the foam generator was started and the foaming pressure adjusted; after steady production was reached, the foam rested for 10 minutes to ensure uniformity and stability.

Because the lightweight carbonated solidified slurry is alkaline, an acidic foaming agent would make the CO_2_ foam dissolve into the slurry, causing quick defoaming and collapse and harming strength; we therefore chose an alkaline foaming agent, and while it reacts readily with CO_2_, the pre-treatment described above tempers that reaction; the resulting CO_2_ foam is largely insoluble in the slurry, so major collapse is avoided and the effect on strength is small.

The rested foam was measured using a graduated cylinder according to the mix proportion and added to the previously mixed slurry. The mixture was stirred for an additional 1 minute to ensure the foam and slurry were uniformly blended and to facilitate carbonation. Finally, the mixed slurry was poured into the specimen molds in three distinct layers. As the foamed lightweight soil possesses self-compacting properties, only slight vibration was needed to fill the molds uniformly. The surfaces of the cast molds were then covered with plastic wrap to prevent moisture evaporation. The cast specimens were placed in a standard curing chamber maintained at a temperature of 20 ± 1°C and a relative humidity of 99% for standard curing. After 48 hours, the specimens were demolded. The demolded specimens were immediately covered with plastic wrap and returned to the curing chamber to continue standard curing until the designated testing age.

### 2.3 Experimental program

The lightweight carbonated solidified slurry is intended for applications such as tunnel-back grouting and subgrade filling, which typically involve rapid construction and low-permeability conditions. Therefore, the UU triaxial test is considered suitable for characterizing its mechanical behavior. Building upon the optimal mix proportions identified in the preliminary orthogonal experimental design by Shao et al. [10], this study investigates the influence of confining pressure, reactive MgO dosage, and CO_2_ foam dosage on the mechanical behavior of the solidified slurry. The experimental program was divided into two series. For all specimens, the dosages of slag powder, calcium carbide slag, anhydrous calcium sulfate, and PAM were fixed at 25%, 7.5%, 5%, and 0.5% of the total waste slurry mass, respectively. All specimens were cured for 28 days and tested under confining pressures of 50, 100, and 200 kPa.

In the first series (investigating MgO dosage), the CO_2_ foam dosage was fixed at 48%, while the reactive MgO dosage was varied at 7%, 10%, 13%, 16%, 19%, and 21%. In the second series (investigating CO_2_ foam dosage), the reactive MgO dosage was maintained at 13%, while the CO_2_ foam volume fraction was varied at 36%, 40%, 44%, 48%, and 52%.

### 2.4 Experimental test methods

In this study, a triaxial apparatus was employed to conduct UU triaxial shear tests on the lightweight carbonated solidified slurry specimens under varying confining pressures, different magnesium oxide dosages, and different CO_2_ foam dosages.

**Fig 3:**
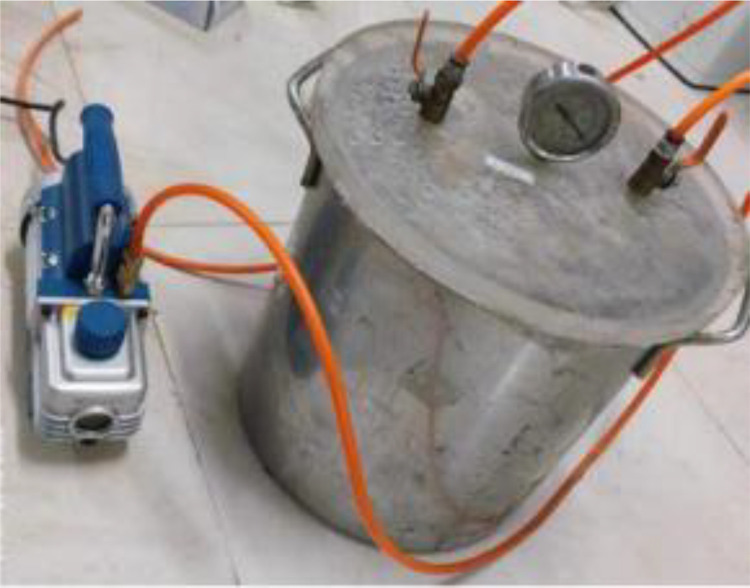
Vacuum saturation apparatus used for triaxial tests.

**Fig 4:**
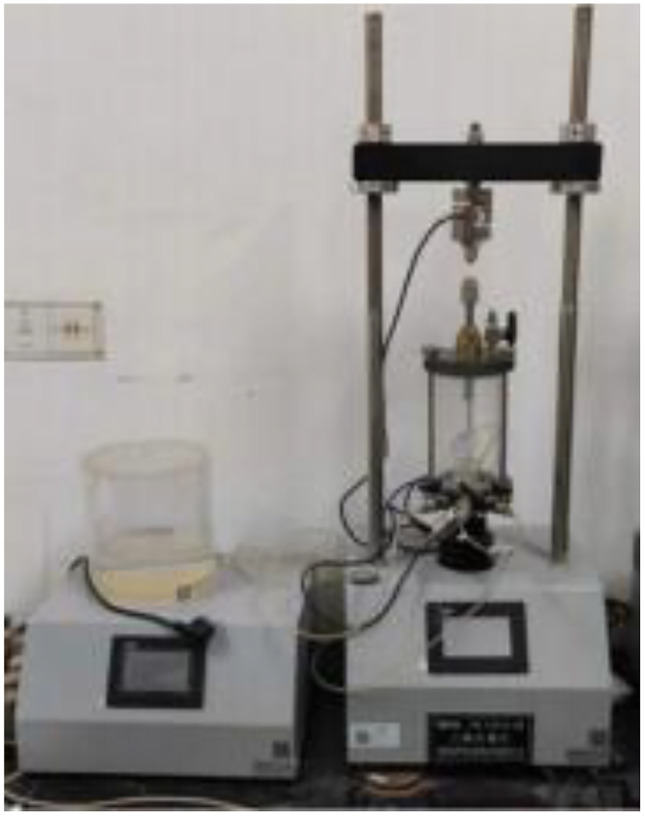
Triaxial compression test apparatus.

Specimens for the triaxial tests were prepared in accordance with the standard [26], with dimensions of 80 mm in height and 39.1 mm in diameter. The prepared specimens were cured in a standard curing chamber. Upon reaching the 28-day curing age, the specimens were retrieved and placed in a desiccator for vacuum saturation as per the standard [27]. The experimental setup is depicted in Fig 3. The desiccator containing the specimens was placed in a water-free vacuum chamber and evacuated. When the vacuum gauge reading reached −1 atm (negative one atmosphere), evacuation was continued for an additional 0.5 hours. After this period, the water inlet pipe was placed into a reservoir of deionized water, and water was slowly introduced while the inlet valve was adjusted to maintain a stable vacuum reading. Once the desiccator was completely submerged, the vacuum pump was turned off, and the water inlet pipe was slowly exposed to the atmosphere. The inlet valve was then opened, allowing atmospheric pressure to facilitate saturation. The specimens were left submerged for 24 hours to ensure full saturation. The triaxial apparatus used for testing is shown in Fig 4.

After the vacuum saturation was complete, the desiccator was disassembled, and the specimens were mounted in the triaxial apparatus as quickly as possible to prevent moisture loss from air exposure. Filter paper and porous stones were placed at both ends of the specimen, which was then enclosed in a latex membrane using a membrane stretcher. The assembly was subsequently installed in the triaxial pressure chamber. In accordance with the standard [28], the test was terminated after the specimen had been sheared to an axial strain 3–5% beyond the point of peak stress. The peak deviatoric stress was defined as the shear strength.

## 3. Experimental results

### 3.1 Effect of reactive magnesium oxide dosage on UU characteristics of lightweight carbonated solidified slurry

This section conducts UU triaxial shear tests to investigate the effect of reactive magnesium oxide dosage on the UU characteristics of lightweight carbonated solidified slurry under different confining pressures by analyzing deviatoric stress-strain curves, softening coefficients, and shear strength.

#### 3.1.1 Effect of reactive magnesium oxide dosage on stress-strain behavior.

**Fig 5:**
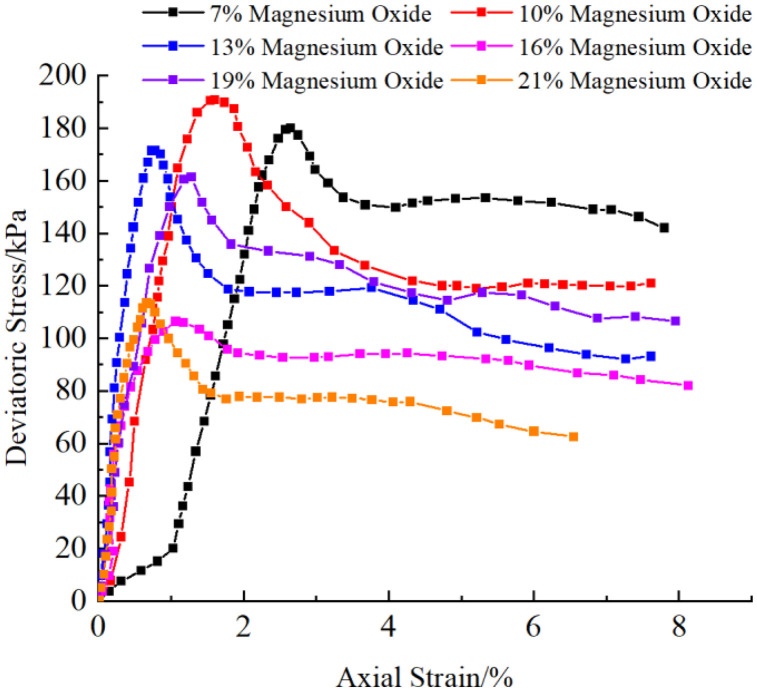
Deviatoric stress-strain curve under 50 kPa confining pressure.

**Fig 6:**
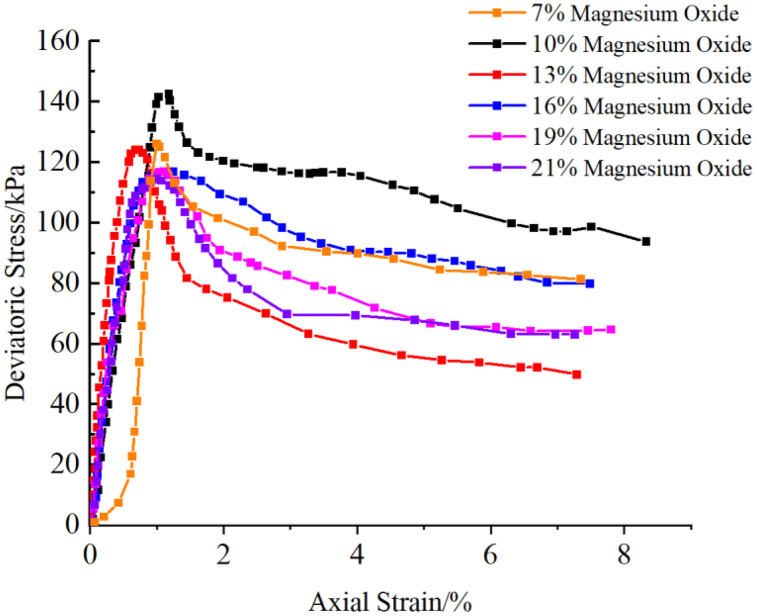
Deviatoric stress-strain curve under 100 kPa confining pressure.

**Fig 7:**
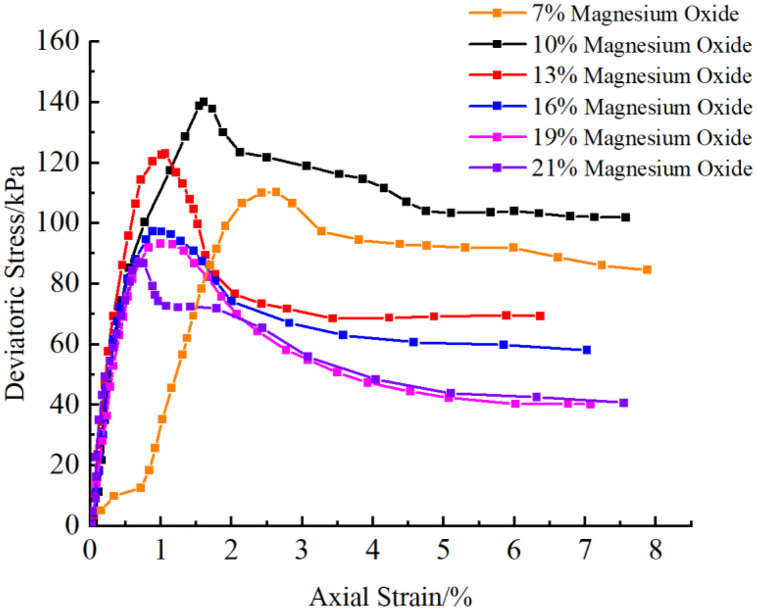
Deviatoric stress-strain curve under 200 kPa confining pressure.

Figs 5–7 show the relationship curves between deviatoric stress and axial strain for specimens with different reactive magnesium oxide dosages at a curing age of 28 days under confining pressures of 50 kPa, 100 kPa, and 200 kPa, where the deviatoric stress is $q=\sigma_{\text{1}}-\sigma_{\text{3}}$.

Fig 5 describes the deviatoric stress-strain curve of specimens with different reactive magnesium oxide dosages at a confining pressure of 50 kPa. As can be seen from the figure, the deviatoric stress increases continuously with the increase of axial strain, and after reaching the peak stress, it drops rapidly and then enters a platform period. The deviatoric stress decreases slowly with the increase of axial strain, showing obvious strain-softening characteristics. The strain softening can be basically divided into four stages [27]: elastic stage, non-linear stage, strain-softening stage, and stress-platform stage.

Lightweight carbonated solidified slurry conforms to the characteristics of porous materials of foamed lightweight soil. Therefore, when axial pressure is applied, the solid material between the internal pores will bear the main pressure. When the axial pressure is small, the lightweight carbonated solidified slurry exhibits elastic characteristics. As the axial stress increases and exceeds the yield strength of the internal solid material, the specimen will undergo brittle failure, and the elastic stage ends. When the strain exceeds the ultimate strain of the specimen, irreversible plastic deformation will occur, and cracks and stress collapse will occur. The damaged solid material will then fill the pores and form a localized densified structure. The localized densified structure can improve a certain bearing capacity, thus leading to a hardening phenomenon. Similar to some elastoplastic materials, if the pressure continues to be applied after the hardening stage, it may lead to structural failure of the lightweight soil, resulting in a softening section, and eventually causing the stress-strain curve to enter a platform stage [29].

The overall deviatoric stress of the specimen shows a trend of first increasing and then decreasing with the increase of the reactive magnesium oxide dosage. When the reactive magnesium oxide dosage is 10%, its peak stress is the highest, at 190.8 kPa. The increase in the dosage of reactive magnesium oxide increases the strength growth rate of the lightweight carbonated solidified slurry, causing the peak stress of the lightweight carbonated solidified slurry to shift to the left with the increase of the magnesium oxide dosage, and the axial strain corresponding to the peak strength becomes smaller. The residual stress of the specimen continuously decreases with the increase of the magnesium oxide dosage. This is because when the dosage of reactive magnesium oxide is too high, the amount of CO_2_ reacting with it is relatively small, leading to insufficient reaction of the reactive magnesium oxide. The hydration product of magnesium oxide, Mg(OH)_2_, has weak cementitious ability and is highly expansive, which leads to an increase in internal pores and a decrease in the strength of the specimen.

Fig 6 shows the deviatoric stress-strain curves of specimens with different reactive magnesium oxide dosages under a confining pressure of 100 kPa, exhibiting typical strain-softening characteristics. Both the peak stress and residual strength of the lightweight carbonated solidified slurry show a trend of first increasing and then decreasing with the increase of the reactive magnesium oxide dosage. When the reactive magnesium oxide dosage is 10%, the peak stress and residual strength of the lightweight carbonated solidified slurry reach their maximum values. This is consistent with the conclusion under a confining pressure of 50 kPa. The increase in the reactive magnesium oxide dosage still improves the early strength growth rate of the slurry.

When the confining pressure is 200 kPa, the deviatoric stress-strain curves for different dosages of reactive magnesium oxide are shown in Fig 7 The dosage of reactive magnesium oxide has a very significant effect on the peak stress and residual strength of the lightweight carbonated solidified slurry.

**Fig 8:**
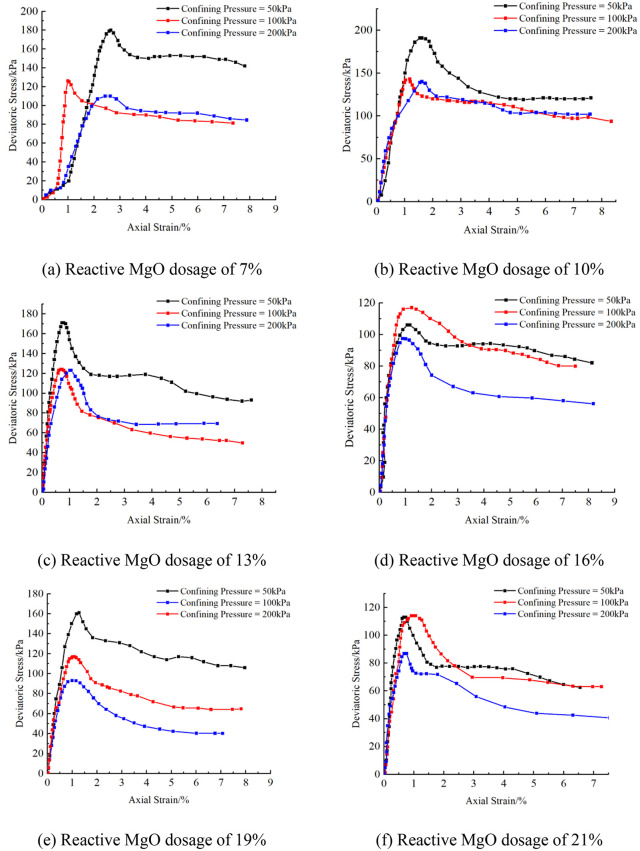
Deviatoric stress-strain curves of specimens under different confining pressures.

Fig 8 shows the deviatoric stress-strain curves of the lightweight carbonated solidified slurry under different confining pressures. Due to the low strength of the lightweight carbonated solidified slurry, it is applied to tunnel excavation backfilling projects and subgrade filling projects that do not have high strength requirements. Therefore, the confining pressures selected for the test are relatively small, at 50 kPa, 100 kPa, and 200 kPa.

As can be seen from the figure, the confining pressure has a significant effect on the peak deviatoric stress and residual strength of the lightweight carbonated solidified slurry. As the confining pressure increases, the peak deviatoric stress of the lightweight carbonated solidified slurry generally shows a downward trend. When the confining pressure is relatively small at 50 kPa, its peak deviatoric stress and residual strength are the highest. This phenomenon is mainly attributed to the specific structural characteristics of the lightweight carbonated solidified slurry [30]. Due to its high porosity, the material relies on a cemented skeleton for strength. Under higher confining pressures (e.g., 200 kPa), the external pressure can exceed the structural yield strength of the porous matrix, causing premature crushing of pore walls and structural collapse before shear failure occurs. This confinement-induced damage leads to stress concentrations and the breakage of cementation bonds, which effectively weakens the material’s skeleton. Consequently, unlike conventional soils, the peak deviatoric stress and residual strength decrease as the confining pressure increases.

#### 3.1.2 Effect of reactive magnesium oxide dosage on softening coefficient.

From the previous deviatoric stress-strain curves, it can be seen that the lightweight carbonated solidified slurry exhibits a distinct strain-softening type, so it is necessary to investigate its softening coefficient. The softening coefficient β is defined as the degree of strain softening of the material, that is, the degree of decrease in the deviatoric stress of the material with the change of axial strain. Its calculation method is as follows:


$$\begin{array}{c} \beta =\frac{q_{\text{p}}-\;q_{\text{r}}}{q_{\text{p}}} \end{array}$$ (1)

where β is material softening coefficient; $q_{\text{p}}$ is peak deviatoric stress (kPa); $q_{\text{r}}$ is residual deviatoric stress (kPa).

From equation (1), it can be known that when the specimen exhibits strain-softening characteristics, β > 0, and when the specimen exhibits strain-hardening characteristics, β < 0. The calculation of the softening coefficient of the lightweight carbonated solidified slurry is shown in Table 5.

**Table 5:** Calculation results of softening coefficient.

Test No.	MgO Dosage (%)	Confining Pressure 50 kPa	Confining Pressure 100kPa	Confining Pressure 200kPa
1	7.00	0.21	0.28	0.16
2	10.00	0.37	0.31	0.27
3	13.00	0.46	0.38	0.35
4	16.00	0.30	0.25	0.24
5	19.00	0.34	0.33	0.30
6	21.00	0.36	0.34	0.32

In order to investigate the effect of different confining pressures on the softening coefficient of the lightweight carbonated solidified slurry, $\beta_{i\text{0}}$ is proposed as the average value of the softening coefficient of the lightweight carbonated solidified slurry with different reactive magnesium oxide dosages under a specific confining pressure. The calculation method is as follows:


$$\begin{array}{c} \beta_{i\text{0}}=\frac{\beta_{i\text{1}}+\beta_{i\text{1}}+\ldots +\beta_{in}}{n} \end{array}$$ (2)

where $\beta_{i\text{0}}$ is average softening coefficient; $\beta_{i\text{n}}$ is softening coefficient under different reactive magnesium oxide dosages;n is number of reactive magnesium oxide dosages, here taken as 6.

**Fig 9:**
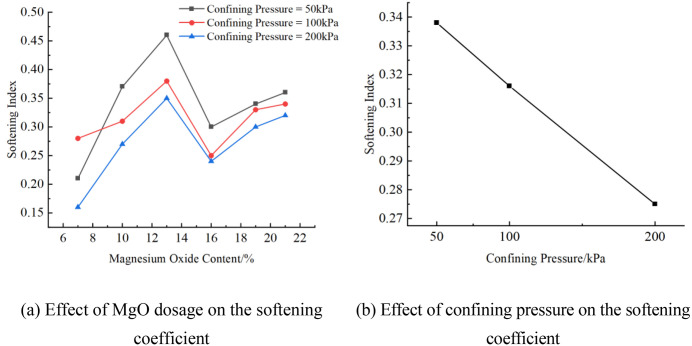
Effect of different reactive magnesium oxide dosages on the softening coefficient.

The effect of the reactive magnesium oxide dosage on its softening coefficient under different confining pressures is shown in Fig 9(b).

Fig 9 shows the effect of different reactive magnesium oxide dosages on the softening coefficient of the lightweight carbonated solidified slurry. From Fig 9(a), it can be seen that the softening coefficient of the lightweight carbonated solidified slurry is generally between 0.2–0.45, and the dosage of reactive magnesium oxide has a greater impact on it. The softening coefficient shows a trend of first increasing and then decreasing with the increase of the reactive magnesium oxide dosage. When the reactive magnesium oxide dosage is 13%, the softening coefficient is the highest, indicating that the deformation capacity of the lightweight carbonated solidified slurry is small and the bearing capacity is high at this time.

From Fig 9(b), it can be seen that the softening coefficient of the lightweight carbonated solidified slurry is negatively correlated with the confining pressure.

#### 3.1.3 Effect of reactive magnesium oxide dosage on shear strength.

Shear strength is a key index of the mechanical performance of lightweight carbonated solidified slurry and depends on slurry type, saturation, and porosity; because this material is highly porous, strength mainly comes from the carbonation products, and the amounts of reactive magnesium oxide (MgO) and CO_2_ foam are especially important. Here we use peak deviatoric stress as the indicator to examine how the MgO dosage shapes the slurry’s shear behavior under different confining pressures.

**Fig 10:**
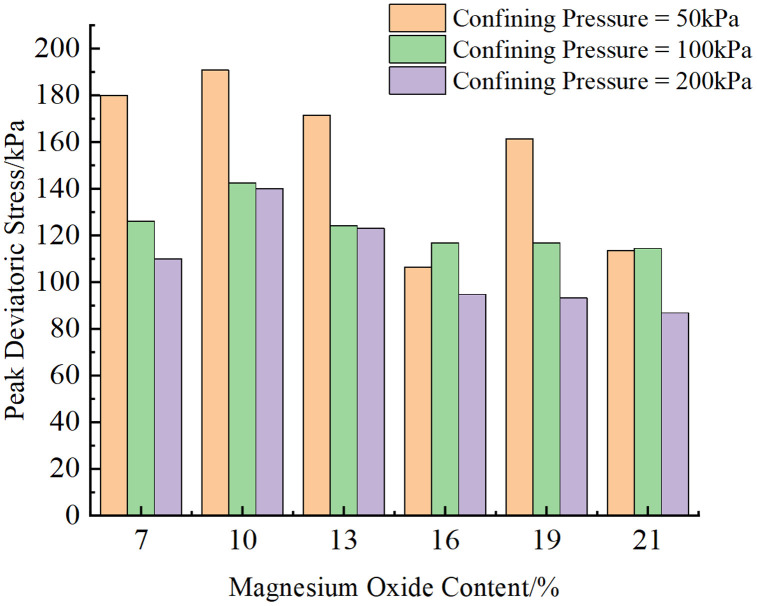
Effect of different MgO dosages on shear strength.

Fig 10 shows how the dosage of reactive magnesium oxide (MgO) affects the shear strength of lightweight carbonated solidified slurry under different confining pressures; shear strength declines as confining pressure increases and is highest at 50 kPa; higher pressures disrupt the overall structure and reduce strength; as MgO dosage rises, the strength first increases and then decreases. When the reactive magnesium oxide dosage is 10%, its shear strength is the highest, indicating that the carbonation reaction is most sufficient at this time, and the carbonation products are the most, thereby providing the shear strength of the lightweight carbonated solidified slurry.

### 3.2 Effect of CO_2_ foam dosage on UU characteristics of lightweight carbonated solidified slurry

This section investigates the effect of CO_2_ foam dosage on the stress-strain of lightweight carbonated solidified slurry under different confining pressures through UU triaxial shear tests, and discusses its strength characteristics, peak deviatoric stress, and shear strength and other mechanical properties, in order to summarize the influence law of CO_2_ foam dosage on the UU shear characteristics of lightweight carbonated solidified slurry.

#### 3.2.1 Effect of CO_2_ foam dosage on stress-strain.

**Fig 11:**
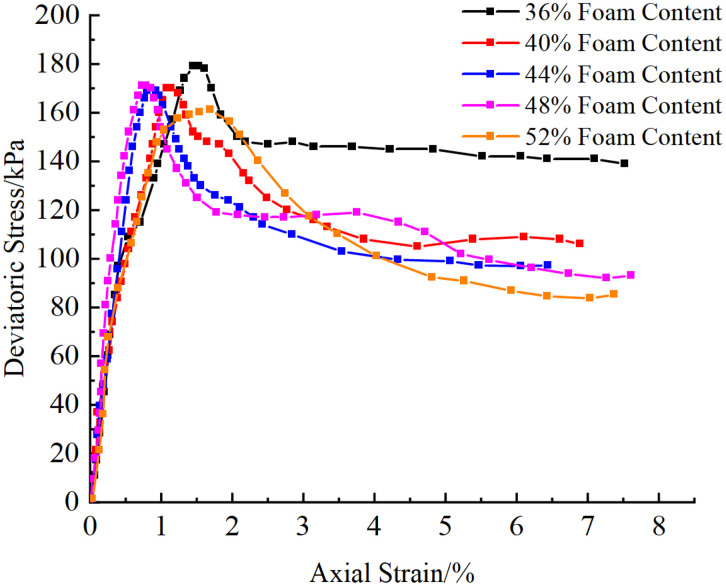
Deviatoric stress-strain curve under 50 kPa confining pressure.

**Fig 12:**
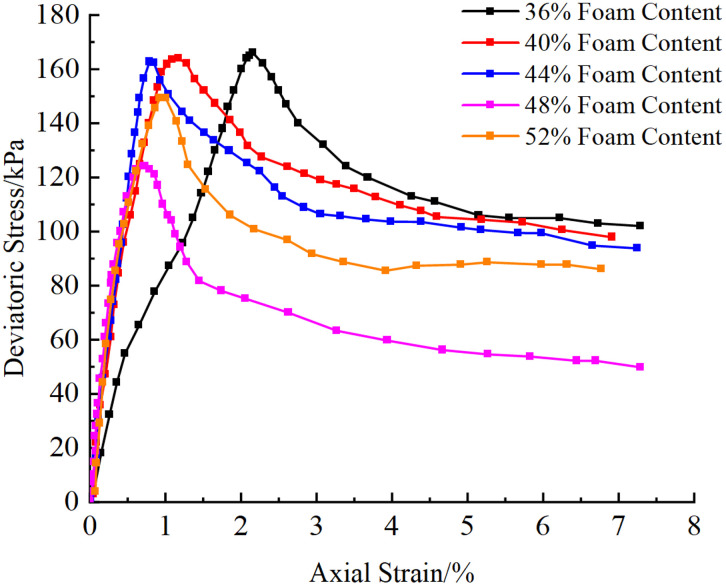
Deviatoric stress-strain curve under 100 kPa confining pressure.

**Fig 13:**
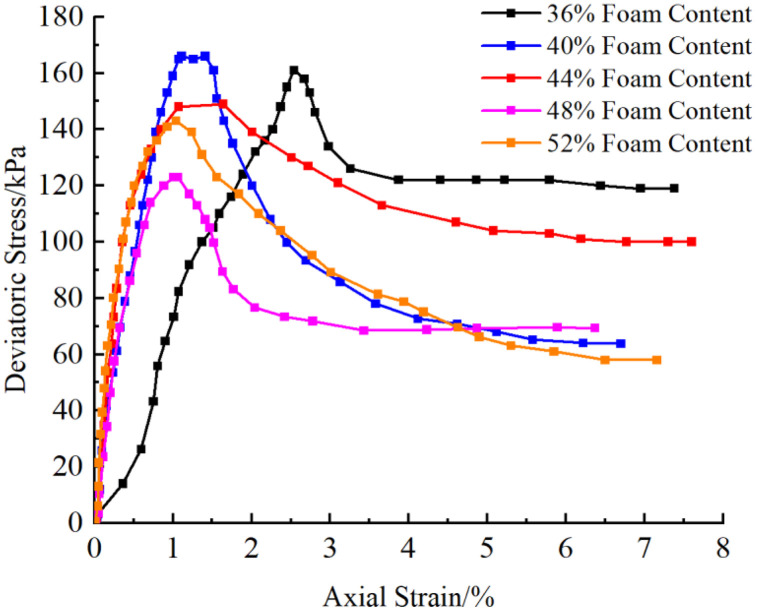
Deviatoric stress-strain curve under 200 kPa confining pressure.

Figs 11–13 show the relationship curves of deviatoric stress and axial strain for specimens with different CO_2_ foam dosages after 28 days of curing under confining pressures of 50 kPa, 100 kPa, and 200 kPa.

Fig 11 shows the deviatoric stress-strain curve of the lightweight carbonated solidified slurry with different CO_2_ foam dosages under a confining pressure of 50 kPa. It can be seen that the specimen as a whole shows obvious strain-softening characteristics, and it begins to decline after reaching the maximum deviatoric stress. The peak deviatoric stress of the lightweight carbonated solidified slurry decreases with the increase of the CO_2_ foam dosage. This is because the incorporation of CO_2_ foam leads to an increase in internal pores and a deterioration of the structure, resulting in a decrease in its peak deviatoric stress. This is consistent with the results of the previous unconfined compressive strength test. When the CO_2_ foam dosage is 36%, the peak deviatoric stress of the lightweight carbonated solidified slurry is the largest, which is 179 kPa under a confining pressure of 50 kPa. At the same time, the residual stress of the specimen also decreases with the increase of the CO_2_ foam dosage. When the CO_2_ foam dosage is 52%, its residual stress is generally around 60 kPa.

Fig 12 describes the deviatoric stress-strain curves of lightweight carbonated solidified slurry with different CO_2_ foam dosages when the confining pressure is 100 kPa. It can be seen that the CO_2_ foam dosage has a significant impact on the peak deviatoric stress and the rate of strength increase of the specimen. As the CO_2_ foam dosage increases, the peak deviatoric stress of the lightweight carbonated solidified slurry gradually decreases, the peak stress point gradually shifts to the left, and the axial strain corresponding to the peak strength becomes smaller

When the specimen confining pressure is relatively large at 200 kPa, its deviatoric stress-strain curves under different CO_2_ foam dosages are shown in Fig 13, and its change law is consistent with that when the confining pressure is small. With the increase of CO_2_ foam dosage, the carbonation reaction of lightweight carbonated solidified slurry is more sufficient, and more carbonation products such as magnesium carbonate are generated. At the same time, it also increases the internal porosity of the specimen, and the bonding force decreases, but it better reduces its wet weight, and the strength of the specimen is improved under the same foam dosage. The residual stress of the specimen decreases with the increase of the CO_2_ foam dosage, but the overall is higher than 60 kPa, which can be well applied in engineering.

**Fig 14:**
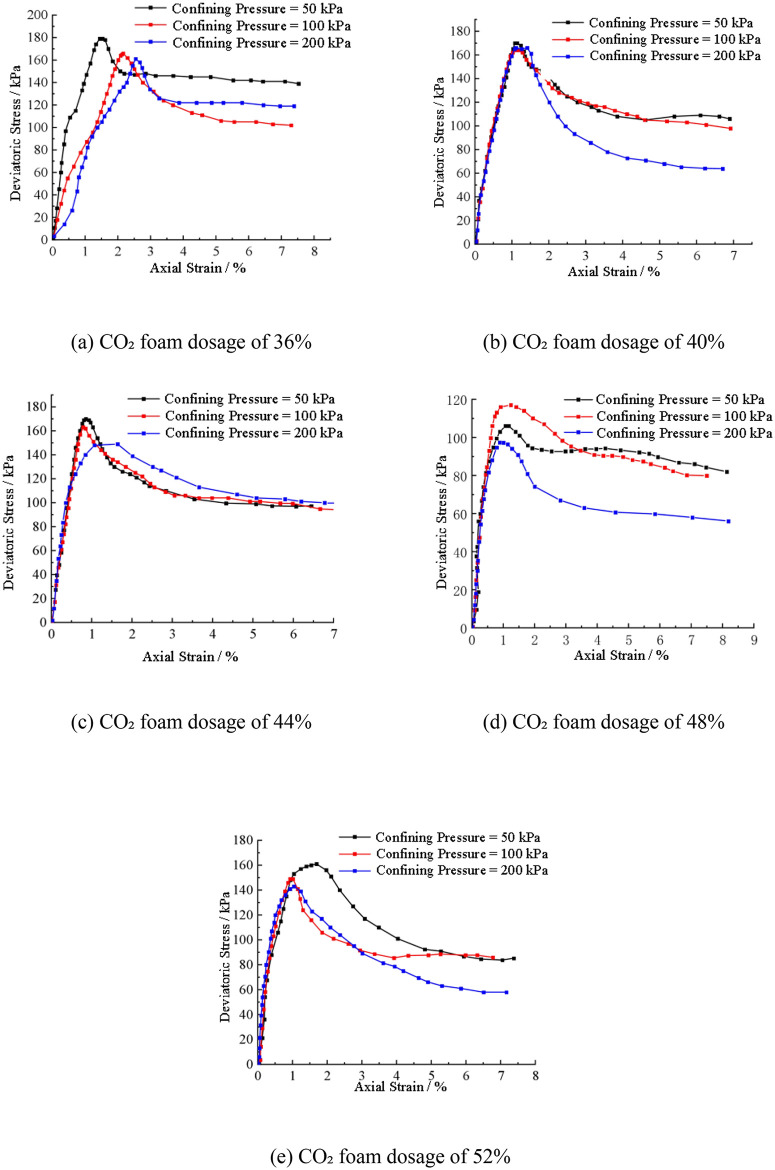
Deviatoric stress-strain curves of specimens under different confining pressures.

Fig 14 shows the deviatoric stress-strain curves of the lightweight carbonated solidified slurry under different confining pressures. It can be seen that in the UU test, the confining pressure has a small effect on the specimen. As the confining pressure increases, the peak deviatoric stress and the residual strength of the specimen generally show a downward trend. When the confining pressure is relatively large at 200 kPa, the internal pores of the specimen are compressed, and the structure of the specimen is destroyed, which is prone to stress concentration, resulting in smaller deviatoric stress and residual strength of the specimen. Moreover, as the CO_2_ foam dosage increases, the internal pores of the specimen increase, and the effect of confining pressure on it is more significant.

#### 3.2.2 Effect of CO_2_ foam dosage on softening coefficient.

**Fig 15:**
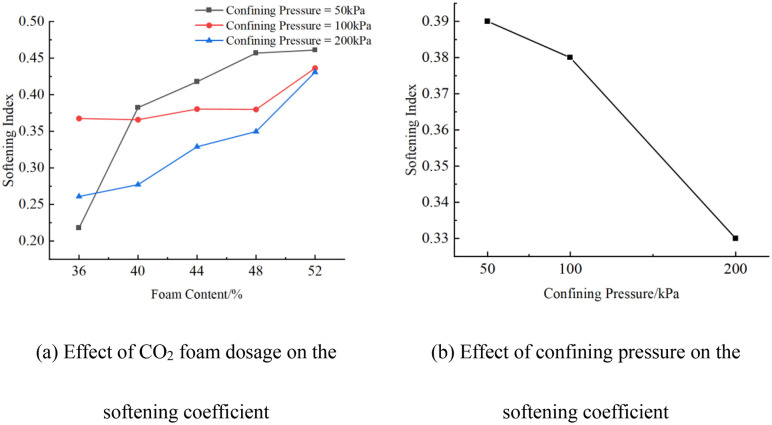
Effect of different CO_2_ foam dosages on the softening coefficient.

The calculation results of the softening coefficient of the lightweight carbonated solidified slurry at different CO_2_ foam dosages are shown in Table 6. The effect of the CO_2_ foam dosage on the softening coefficient of the lightweight carbonated solidified slurry under different confining pressures is shown in Fig 15.

**Table 6:** Calculation results of softening coefficient.

Test No.	MgO Dosage (%)	Confining Pressure 50 kPa	Confining Pressure 100 kPa	Confining Pressure 200 kPa
O (1)	36.00	0.22	0.37	0.26
O (2)	40.00	0.38	0.37	0.28
O (3)	44.00	0.42	0.38	0.33
O (4)	48.00	0.46	0.38	0.35
O (5)	52.00	0.46	0.44	0.43

From Fig 15(a), it can be seen that the softening coefficient of the lightweight carbonated solidified slurry is greatly affected by the CO_2_ foam dosage. When the foam dosage is 36−52%, the overall range of its softening coefficient is between 0.20–0.50 and shows an increasing trend with the increase of the CO_2_ foam dosage. This is because when the CO_2_ foam dosage is high, the internal porosity of the lightweight carbonated solidified slurry is high, the structure is relatively loose, and the volume compressibility of the specimen is better. When subjected to the corresponding axial stress, its volume change is larger, and it can withstand larger deformation.

From Fig 15(b), it can be seen that the softening coefficient of the lightweight carbonated solidified slurry is negatively correlated with the confining pressure. When the confining pressure is high, the specimen is squeezed by the outside, resulting in poor compressibility. When subjected to the same axial stress, its deformation is limited, resulting in smaller volume changes, and the deformation that can be withstood is smaller than that under low confining pressure.

#### 3.2.3 Effect of CO_2_ foam dosage on shear strength.

From the previous tests, it can be known that the strength of the lightweight carbonated solidified slurry is greatly affected by the CO_2_ foam dosage. Therefore, this section uses the peak deviatoric stress as an indicator to study the effect of the CO_2_ foam dosage on the shear characteristics of the lightweight carbonated solidified slurry under different confining pressures. Previous studies on the triaxial shear mechanical properties of reinforced foamed lightweight soil [31] and the UU characteristics of soil samples [32] have generally concluded that there is an exponential function relationship between the peak deviatoric stress and the dosage of the sample. Therefore, this section uses a Quadratic function model, removing the more discrete data points, to fit the law between the peak deviatoric stress of the lightweight carbonated solidified slurry and the CO_2_ foam dosage. The basic formula of this function is:


$$\begin{array}{c} qr=q+zS_{v}+p\overset{\text{2}}{\underset{v}{S}} \end{array}$$ (3)

where *qᵣ* is peak deviatoric stress of lightweight carbonated solidified slurry (kPa); *q, z, p* is fitting function parameters; *S*_*v*_ is CO_2_ foam dosage of lightweight carbonated solidified slurry (%).

**Fig 16:**
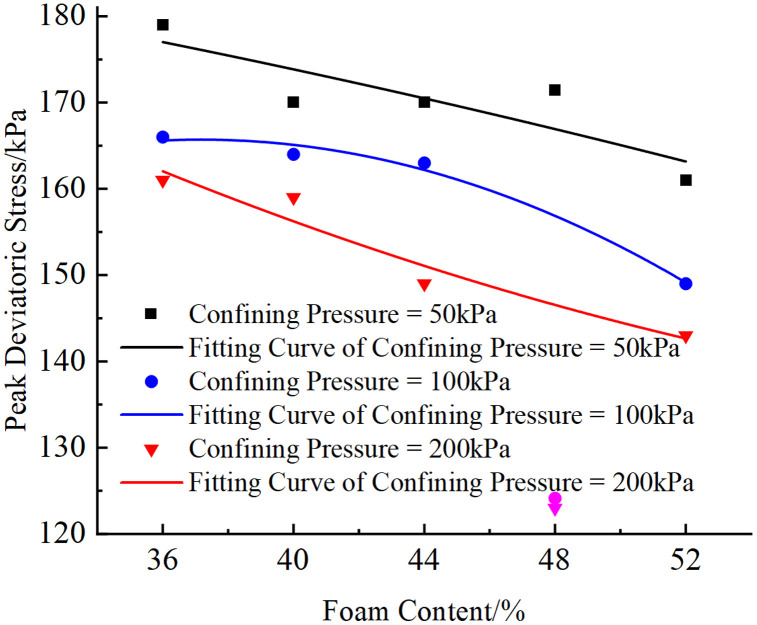
Effect of different CO_2_ foam dosages on shear strength.

Fig 16 shows the fitting curve between the peak deviatoric stress of the lightweight carbonated solidified slurry and the CO_2_ foam dosage under different confining pressures, and Table 7 shows the fitting parameter table.

**Table 7:** Fitting parameter table.

Confining Pressure (kPa)	a	b	c	R^2^
50	196.44	−0.32	−0.06	0.73
100	61.68	5.59	−0.08	0.99
200	242.87	−2.96	−0.02	0.94

The fitting results show that there is a significant exponential relationship between the CO_2_ foam dosage and shear strength of the lightweight carbonated solidified slurry under different confining pressures, and its shear strength shows a decreasing trend with the increase of the CO_2_ foam dosage. The fitting parameters a, b, and c together characterize the reduction in shear strength of the lightweight carbonated solidified slurry with the increase of the CO_2_ foam dosage. The trend of its shear strength change is consistent with the trend of unconfined compressive strength change obtained from previous tests. With the increase of CO_2_ foam dosage, the carbonation products of the lightweight carbonated solidified slurry increase, but the porosity increases, leading to a decrease in its shear strength.

From the fitting curve, it can be known that the shear strength of the lightweight carbonated solidified slurry shows a downward trend with the increase of the confining pressure, which indicates that when the confining pressure of the lightweight carbonated solidified slurry is required to be high in engineering, the CO_2_ foam should not be excessively mixed. At present, it is not perfect to predict the shear strength of lightweight carbonated solidified slurry through the CO_2_ dosage. This formula (3) can predict the shear strength of lightweight carbonated solidified slurry under different stress conditions in engineering, which has practical engineering significance.

## 4. Constitutive model study

This section establishes a constitutive model suitable for lightweight carbonated solidified slurry, analyzes the previous test data, determines the required parameters of the model, and compares the experimentally measured deviatoric stress-strain curve with the model curve to analyze the accuracy of the model.

### 4.1 Constitutive model establishment

Solidified soil is a complex material affected by various factors such as water content, porosity, and saturation. According to previous research, there is currently no constitutive model suitable for all soils and solidified soils [33]. In 1970, Duncan et al. proposed the Duncan-Chang model based on the idea that the stress-strain curve of solidified soil can be fitted with a hyperbola, which was proposed by Kondner. The Duncan-Chang model is one of the most widely used nonlinear elastic models and can be used for stress-strain research of soil and solidified soil.

Duncan-Chang model can reflect the nonlinear stress-strain characteristics of soil, and its expression is:


$$\begin{array}{c} \sigma_{\text{1}}-\sigma_{\text{3}}=\frac{\varepsilon_{\text{1}}}{a+b\varepsilon_{\text{1}}} \end{array}$$ (4)

where $\sigma_{\text{1}}$ is maximum principal stress (kPa); $\sigma_{\text{3}}$ is minimum principal stress (kPa); $\varepsilon_{\text{1}}$ is axial strain (%); a, b: fitting coefficients.

The Duncan-Chang model represented by Equation (4) can also be expressed as:


$$\begin{array}{c} \frac{\varepsilon_{\text{1}}}{\sigma_{\text{1}}-\sigma_{\text{3}}}=a+b\varepsilon_{\text{1}} \end{array}$$ (5)

**Fig 17:**
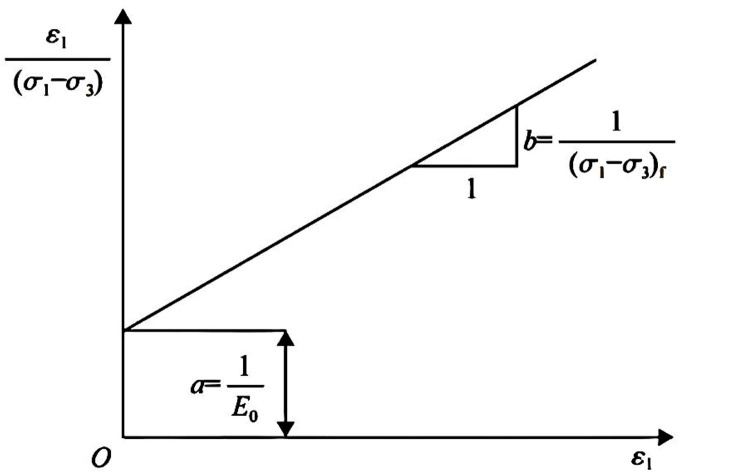
Schematic diagram of the Duncan-Chang model relationship.

From equation (5), it can be found that when the axial strain $\varepsilon_{\text{1}}\;<\;\text{1}\%$, $\varepsilon_{\text{1}}\;/\;(\sigma_{\text{1}}-\sigma_{\text{3}})\;$ and $\varepsilon_{\text{1}}$ have a linear relationship. Where *a* is the intercept of the straight line and *b* is the slope of the straight line, as shown in Fig 17.

**Fig 18:**
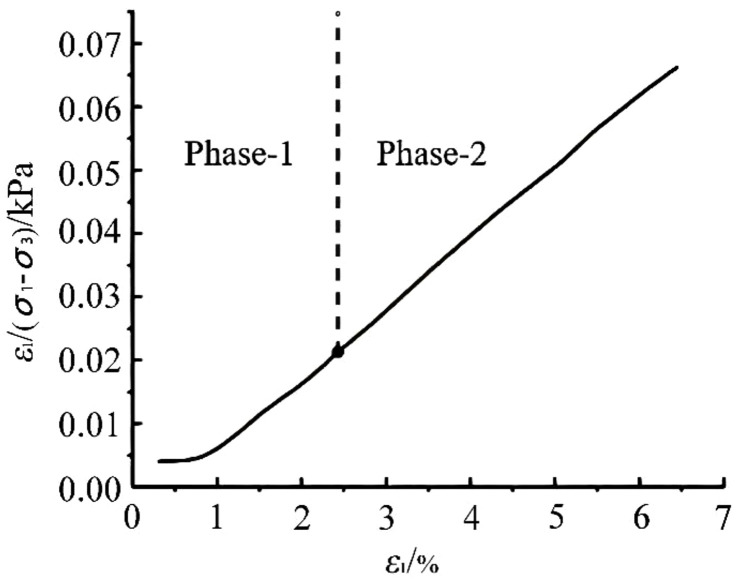
Relationship curve of lightweight carbonated solidified slurry $\varepsilon /(\sigma_{1}-\sigma_{3})-\varepsilon$.

However, the traditional Duncan-Chang model is only applicable to the stress-strain relationship where the $\varepsilon /(\sigma_{\text{1}}-\sigma_{\text{3}})-\varepsilon$ curve is a single straight line, and it cannot well handle the situation where the $\varepsilon /(\sigma_{\text{1}}-\sigma_{\text{3}})-\varepsilon$ curve is segmented. Therefore, it is necessary to modify it. According to the division results in Fig 18, the stress-strain relationship of the lightweight carbonated solidified slurry is divided into two stages, which are analyzed and processed separately.

As can be seen from Fig 18, when the lightweight carbonated solidified slurry $\varepsilon /(\sigma_{\text{1}}-\sigma_{\text{3}})-\varepsilon$ curve is in the first stage, the deformation produced by the soil is elastoplastic deformation. At this time, the stress changes non-linearly with the increase of strain. When the lightweight carbonated solidified slurry $\;\varepsilon /(\sigma_{\text{1}}-\sigma_{\text{3}})-\varepsilon \;$ curve is in the second stage, the specimen structure is damaged, and the deformation is mainly plastic deformation. The corresponding $\;\varepsilon /(\sigma_{\text{1}}-\sigma_{\text{3}})-\varepsilon$ curve is a straight line. In order to better describe the stress-strain curve of the lightweight carbonated solidified slurry, this section establishes a model in stages:

(1) First stage

A statistical damage constitutive model [31], which is based on the statistical distribution of material strength, can well describe the elastoplastic changes of the specimen, which can be expressed as:


$$\begin{array}{c} \left(\sigma_{\text{1}}-\sigma_{\text{3}}{)}_{\text{1}}=E\varepsilon_{\text{1}}\cdot \text{exp}[-\frac{\text{1}}{p}(\frac{\varepsilon_{\text{1}}}{\varepsilon_{pk}}{)}^{P}\right] \end{array}$$ (6)

$$\begin{array}{c} p=\frac{\text{1}}{\text{ln}\left(\frac{E\cdot \varepsilon_{pk}}{\sigma_{pk}}\right)} \end{array}$$ (7)

where E is elastic modulus (kPa); $\varepsilon_{\text{pk}}$ is axial strain corresponding to the peak stress (%); p is shape parameter, which can be calculated according to equation (7); $\varepsilon_{\text{1}}$ is axial strain in the first stage (%); $\sigma_{\text{pk}}$ is peak stress (kPa).

(2) Second Stage

As can be seen from Fig 18, the slope of the straight line in the second stage of the lightweight carbonated solidified slurry is larger, and the intercept of the soil mass is negative, that is, the initial shear modulus of the soil mass is negative. Zhang et al. [34] proposed a modified Duncan-Chang model applicable to strongly structured soil based on the Duncan-Chang model, which can better reflect the changes in the second stage. According to the conventional triaxial test stress-strain relationship curve, the corresponding strain ε_125_ when the specimen enters the second stage can be obtained. This value is a constant. Then the stress-strain relationship of the soil in the second stage can be expressed as:


$$\begin{array}{c} (\sigma_{\text{1}}-\sigma_{\text{3}}{)}_{\text{2}}=\frac{\varepsilon_{\text{2}}+\varepsilon_{\text{1}\text{2}s}}{m+n(\varepsilon_{\text{2}}+\varepsilon_{\text{1}\text{2}s})}\leftarrow \end{array}$$ (8)

Equation (4.8) can also be rewritten as:


$$\begin{array}{c} (\sigma_{\text{1}}-\sigma_{\text{3}}{)}_{\text{2}}=\text{1}/(\left(\frac{\text{m}}{\varepsilon_{\text{2}}+\varepsilon_{\text{12}s}}\right)+\text{n})(\sigma_{\text{1}}-\sigma_{\text{3}}{)}_{\text{2}}=\frac{\text{1}}{\frac{m}{\varepsilon_{\text{2}}+\varepsilon_{\text{12}s}}+n} \end{array}$$ (9)

$$\begin{array}{c} (\sigma_{\text{1}}-\sigma_{\text{3}}{)}_{ult\text{2}}=\frac{\text{1}}{n} \end{array}$$ (10)

where $\varepsilon_{\text{2}}$ is axial strain in the first stage (%); $\varepsilon_{\text{1}\text{2}s}\;$ is corresponding axial strain when entering the second stage (%); *m* is corresponding intercept of the soil mass; *n* is calculation method is shown in equation (10); $(\sigma_{\text{1}}-\sigma_{\text{3}}{)}_{ult\text{2}}$ is the theoretical ultimate deviatoric stress (kPa).

### 4.2 Reactive magnesium oxide dosage model

By substituting the previous experimental results into equations (6) – (10) for calculation, the model parameters of the reactive magnesium oxide dosage of the lightweight carbonated solidified slurry can be obtained, as shown in Table 8.

**Table 8:** Reactive magnesium oxide dosage model parameters.

Test No.	Confining Pressure (kPa)	*m*	*E*	*n*	$\varepsilon_{12s}$	*p*
1	50	−0.0028	99.0000	0.0074	3.70	2.6505
	100	−0.0073	322.0000	0.0125	1.92	1.0738
	200	−0.0090	67.3000	0.0125	3.27	2.5216
2	50	−0.0050	165.6271	0.0090	2.50	3.0139
	100	−0.0130	122.7592	0.0129	1.23	64.8983
	200	−0.0068	243.4952	0.0097	2.70	0.9712
3	50	−0.0175	500.5521	0.0139	1.47	1.2053
	100	−0.0250	471.3836	0.0200	1.52	0.9849
	200	−0.0183	285.9502	0.0167	2.04	1.1715
	50	−0.0212	432.1023	0.0160	1.92	0.6825
4	100	−0.0151	263.4278	0.0139	2.18	0.9740
	200	−0.0152	323.5082	0.0188	1.90	0.9265
	50	−0.0096	236.2893	1.9700	0.01	1.5902
5	100	−0.0147	228.5677	0.0153	1.94	1.2986
	200	−0.0345	217.1063	0.0236	2.07	1.1882
6	50	−0.0085	442.8403	0.0160	1.76	1.0470
	100	−0.0147	270.0000	0.0157	2.30	1.1633

**Fig 19:**
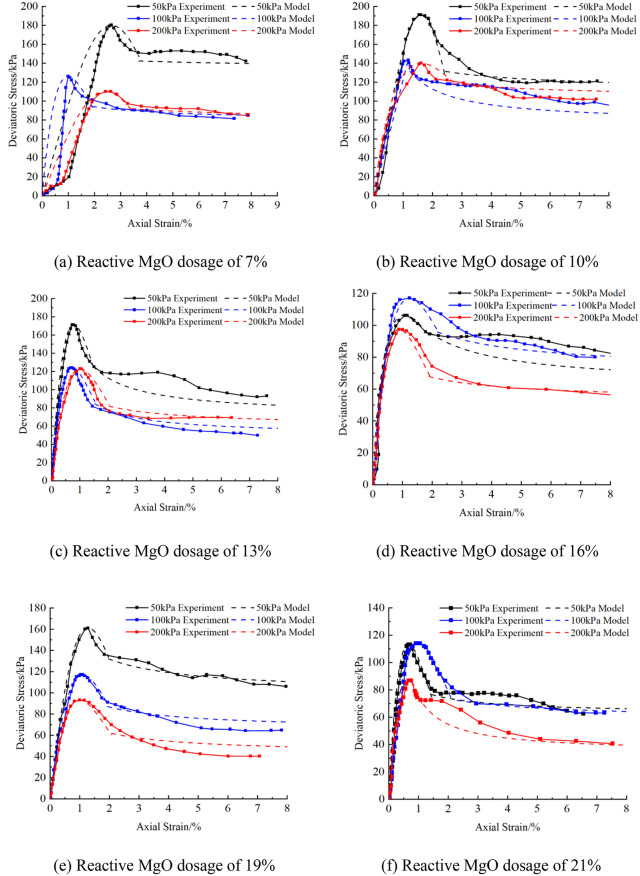
Comparison between model and experimental results.

In the previous UU test of lightweight carbonated solidified slurry, the confining pressures were set to 50 kPa, 100 kPa, and 200 kPa. The deviatoric stress-strain curves of the lightweight carbonated solidified slurry with different reactive magnesium oxide dosages will be fitted with the model in this section and placed in the same coordinate system for comparison to verify the accuracy of the model established in this section. The comparison diagram is shown in Fig 19.

From the comparison in Fig 19, it can be known that in most cases, the calculation results of the constitutive model are in good agreement with the experimental data, with high accuracy, indicating that the constitutive model is suitable for lightweight carbonated solidified slurry. When the reactive magnesium oxide dosage is high, the elastic modulus is large, the model curve fits well, and the fitting accuracy is high. When $\varepsilon \;=\;\varepsilon_{\text{1}\text{2}\text{5}}$, there is a slight discrepancy between the model curve and the experimental data curve. This is because the damage model of the first stage cannot well reflect the strain-softening stage, but it is basically close to the experimental value in the elastic stage and the non-linear stage.

### 4.3 CO_2_ foam dosage model

By substituting the previous test results into equations (6) – (10) for calculation, the model parameters of the CO_2_ foam dosage of the lightweight carbonated solidified slurry can be obtained, as shown in Table 9.

**Table 9:** CO_2_ foam dosage model parameters.

Test No.	Confining Pressure (kPa)	*m*	*E*	*n*	$\varepsilon_{12s}$	*p*
O (1)	50	−0.0016	197.5174	0.0072	2.09	2.1276
	100	−0.0130	93.0000	0.0105	2.80	5.2433
	200	−0.0023	78.0000	0.0084	3.26	4.8200
	50	−0.0055	243.0000	0.0100	1.73	2.3030
O (2)	100	−0.0082	249.5961	0.0105	1.85	1.9757
	200	−0.0204	190.0000	0.0166	1.48	4.1772
	50	−0.0060	330.0000	0.0105	1.45	1.9738
O (3)	100	−0.0057	265.0000	0.0105	1.05	4.0368
	200	−0.0087	312.0000	0.0104	3.50	0.8105
	50	−0.0175	500.5521	0.0139	1.47	1.2053
O (4)	100	−0.0250	471.3836	0.0200	1.52	0.9849
	200	−0.0183	285.9502	0.0167	2.04	1.1715
	50	−0.0176	259.0000	0.0125	3.90	0.9998
O (5)	100	−0.0047	369.3520	0.0117	1.95	1.1988
	200	−0.0407	572.1811	0.0200	3.60	0.7013

**Fig 20:**
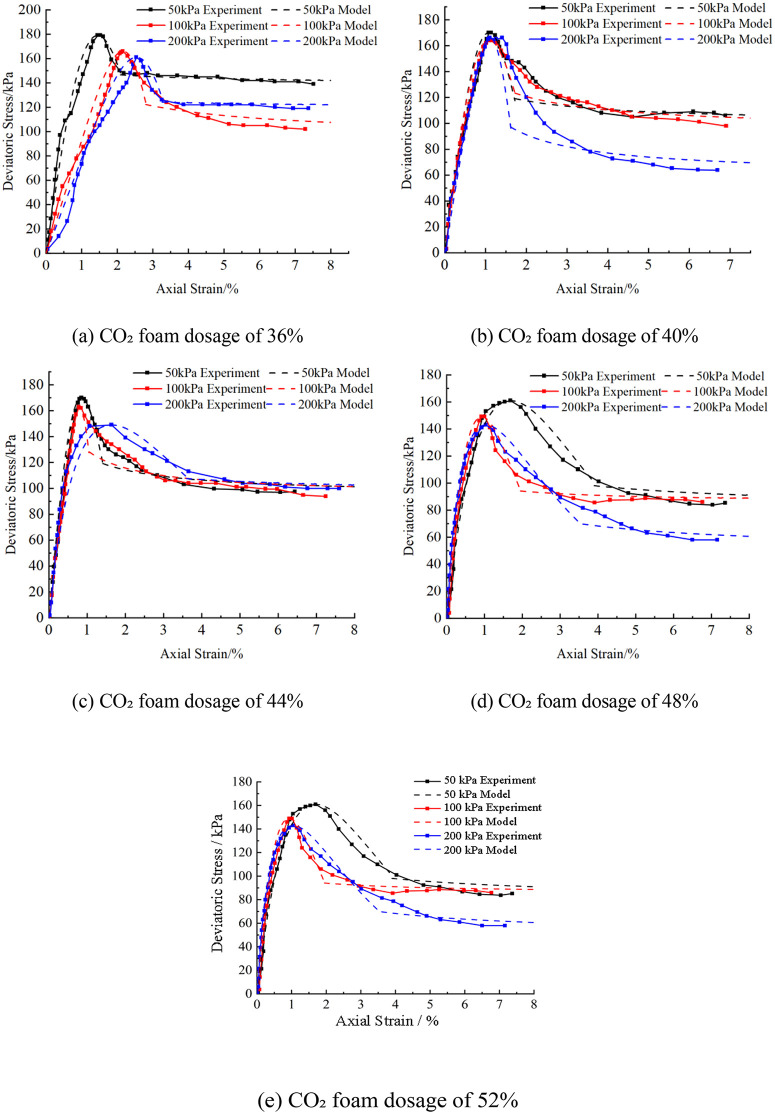
Comparison between model and experimental results.

The deviatoric stress-strain curves of the lightweight carbonated solidified slurry with different CO_2_ foam dosages will be fitted with the model in this section and placed in the same coordinate system for comparison to verify the accuracy of the model established in this section. The comparison diagram is shown in Fig 20.

As can be seen from Fig 20, the data from this constitutive model fits well with the experimental data, which can more accurately reflect the deviatoric stress-strain curve of the lightweight carbonated solidified slurry under different confining pressures. When the specimen deformation reaches the interface between the first and second stages, the model and the measured data have some discrepancies but are not large. Overall, the model can better reflect the stress-strain trend of the lightweight carbonated solidified slurry, and the gap between the model curve and the data-measured curve is small, which indicates that the model has high accuracy and good fitting effect.

## 5. Conclusions

This study investigated the influence of reactive magnesium oxide (MgO) dosage and CO_2_ foam content on the deviatoric stress–strain curves, softening coefficients, and shear strength of lightweight carbonated solidified slurry under various confining pressures, based on triaxial compression tests. A constitutive model was established utilizing a damage model and the modified Duncan–Chang framework, which holds practical significance for engineering applications. The main conclusions are as follows:

(1) Reactive MgO dosage strongly shapes the deviatoric stress–strain curves of lightweight carbonated solidified slurry, which show strain-softening behavior; as dosage increases, the peak deviatoric stress, residual stress, and shear strength first rise then fall, and all three decrease as confining pressure grows, with the highest peak and residual stresses at 13% MgO. The softening coefficient follows a rise–fall–rise pattern with MgO dosage, peaking at 13%, and it is negatively correlated with confining pressure.
(2) Both the peak deviatoric stress and the residual stress drop as CO_2_ foam content increases and as confining pressure rises; the softening coefficient is strongly affected by foam content—growing with more foam—and it shows a negative correlation with confining pressure; shear strength likewise decreases as foam content increases; to capture these relations under different stress states, an exponential function was fitted, offering practical engineering value.
(3) The (UU experimental data of the lightweight carbonated solidified slurry were analyzed and processed using a model based on damage theory and the modified Duncan–Chang framework. A comparison between the model-predicted curves and the measured experimental curves demonstrates that the proposed model can effectively reflect the stress–strain evolution of the lightweight carbonated solidified slurry. The model exhibits high precision and a good fitting performance.

While this study successfully establishes the baseline static mechanical behavior and a damage-based constitutive model, further research is required to fully bridge the gap between micro-mechanisms and long-term engineering performance. Future work will focus on two key aspects: first, quantitative microstructural characterization (via XRD and SEM) will be conducted to visually verify the formation of hydrated magnesium carbonates and directly correlate pore structure evolution with the observed strain-softening behavior; second, long-term durability assessments, specifically cyclic loading tests and wet-dry cycle simulations, will be performed to evaluate the fatigue life and strength retention of the carbonated solidified slurry under complex environmental and service conditions.
